# Health Tract, No. 148

**Published:** 1863-07

**Authors:** 


### SUMMER DRINKS.

In passing along Nassau street, near the Post-Office, any hot summer's day, there may be seen a sign on which is written "Iced Whisky." The newspapers abound in recipes for making a great variety of cooling drinks for the summer-time. A corner of the Post-Office of the great city of New-York is hired to a man for a few dollars a year, who has perhaps a dozen different kinds of "cooling" drinks, patronized mainly by store-boys, and paid for, in too many cases, by pennies filched from their employers. The cigars and tobacco sold at the same place are, doubtless, paid for in the same way, and are as cooling as the "iced whisky" near the office of the *Evening Post.* The absurdity of any thing having a cooling effect on the human system which contains a particle of alcohol, whether cognac, lager, or cider, need not be remarked on. If these things are cooling, how comes it that they are never by any chance offered in summer-time without ice, or iced water? It is greatly to be regretted that the United States Government, or the Postmaster of New-York, should, for a few dollars a year, be "*particeps criminis*" in making spendthrifts, drunkards, and thieves of store-boys, who are generally, perhaps, sent from the country with a certain degree of purity of character, tenderness of conscience, and constitutional vigor, with a view of becoming merchant-princes and useful men. A similar crime against society is committed, inadvertently no doubt, by our family and religious newspapers, in sending out their directions for making "cooling drinks for summer," in the shape of root-beers, lemonades, mulled wine, and the like. Whatever tempts to drink liquids, even cold water in hot weather, endangers health and life itself. Even the iced Croton at our dinner-tables and at the public schools (by the contributions of the scholars) is wholly injurious to the general health and most pernicious to the teeth. It is not true that soda-water even is harmless. A boy who takes a glass to-day in the corner of the Post-Office will feel like doing it to-morrow, and in less than a week the desire for it will come the instant he gets in sight of that famous corner; after a while he will want another glass in the afternoon; later on, it will be lemonades, into which the venders have already begun to introduce coloring matters, syrups, wines, and "old Bourbon." These are the beginnings of the end of a drunkard's dreadful fate. If a man is really thirsty, there is nothing more delicious, nothing which is more gratefully and perfectly satisfying, than a glass of cool water, with the advantage of its costing nothing, and besides leads to no bad habits. The men in glass manufactories, where the heat is fearful, drink water only, and that not iced, and remain healthy and vigorous. Field-hands on cotton and sugar plantations find a wholesome drink in a mixture of molasses and water; this is a safe drink for harvesters; so also is "buttermilk," it being not only cooling and nutritious, but otherwise healthful as a liver stimulant.

